# Discrepancy between high non-verbal intelligence and low accuracy at reading emotional expressions in the eyes reflects the magnitude of social–emotional difficulties in autism

**DOI:** 10.1007/s00406-022-01471-z

**Published:** 2022-08-18

**Authors:** Nouchine Hadjikhani, Martyna Galazka, Tal Kenet, Robert Joseph, Jakob Åsberg Johnels

**Affiliations:** 1 Harvard/MGH Martinos Center for Biomedical Imaging, Boston, MA USA; 2 Gillberg Neuropsychiatry Centre, University of Gothenburg, Gothenburg, Sweden; 3 Boston University School of Medicine, Boston, MA USA

**Keywords:** Autism, Social understanding, Eye contact

## Abstract

Many so-called “high functioning” autistic individuals struggle with daily living skills, and have poorer than expected adult outcomes in employment, relationships, and quality of life. Significant discrepancies between non-verbal intelligence and emotional processing can be observed in autism, but the role of the magnitude of this gap in achieving potential psychosocial outcome is not known. Here, we show in a large group of participants (*n* = 107), that only among those with an autism diagnosis (*n* = 33), the gap between non-verbal intelligence (as measured by Raven’s matrices) and the ability to perform the Reading the Mind in the Eyes test significantly predicts self-perceived emotional/social difficulties as assessed by the Empathy Quotient. Our results suggest that it is specifically the magnitude of the gap between (high) levels of abstract reasoning skills and poor proficiency in reading emotions expressed by the eyes that predicts self-perceived difficulties in emotional and social interactions among adults with autism. A better understanding of the underlying causes of the discrepancy between potential and actual psychosocial outcomes is the first step toward developing the most appropriate support for this vulnerable population, and our study offers some potentially important insights in this regard.

**Supplementary Information:**

The online version contains supplementary material available at 10.1007/s00406-022-01471-z.

## Introduction

Autism is a lifelong condition, and children with autism spectrum disorder (ASD) grow into adults with ASD. While many individuals with ASD require lifelong support, those with average and above-average general intelligence (IQ), i.e., without intellectual disability (referred to from now on as ASD_noID_), are generally considered to be “high functioning”. However, despite terminology, many ASD_noID_ individuals do not have adult outcomes commensurate with their IQ. Several studies report that many so-called “high functioning” ASD individuals show a gap between cognitive skills and adaptive behavior, and struggle with everyday functions (e.g., [1, 2, 8, 11, 13–17, 20, 26, 31]). For example, a large number of studies on occupational activities in adults with ASD (reviewed in [16]) show that a considerable proportion of adults with above-average IQ are unemployed or underemployed, that salary of employed adults with ASD is significantly lower than for adults with other disabilities, and that despite average levels of education, ASD_noID_ adults have high rates of financial dependency [28], and poorer quality of life (QoL) [22, 25].

Causes of the poorer than expected outcomes in a proportion of autistic individuals who do not have any intellectual disability are still not well understood. One of the underlying mechanisms could have its origins in the greater difficulty experienced when deciphering emotions expressed by others—an ability that largely relies on extracting information from the eye region. Yet, it is well known that many autistic individuals tend to avoid looking at eyes [29, 32].

The Reading the Mind in the Eyes Test (RMET) [4] measures the ability to understand others’ emotional state based on the picture of the eyes of an actor. It has been shown that autistic individuals have worse performance than typical controls on the RMET ([3, 4]). It has also been shown that RMET scores are positively related to emotional empathy [6]. The Empathy Quotient (EQ) is a self-measure of cognitive empathy, emotional reactivity, and social skills [18], and low RMET accuracy has been associated with low EQ scores [6, 9].

What has not been examined is the experience of falling relatively much below on RMET relative to one’s own general cognitive capacity. Interestingly, research on another neurodevelopmental difficulty (dyslexia) has shown that the dyslexic impairment is seen as particularly disabling for those individuals with high general cognitive skills, potentially due to the discrepancy between reading attainment and the higher personal and external expectations for this population [10]. In autism, it is possible that a similar discrepancy between high cognitive skills and low social/emotional skills results in higher self-perceived disability, and greater challenges in daily living.

Here, we examined performance on the RMET together with performance on Raven’s Progressive Matrices (RPM) [27], a non-verbal test of abstract reasoning in a group of autistic individuals (*n* = 33) and typical controls (*n* = 74). We used the Empathy Quotient (EQ) test to assess self-perceived everyday social–emotional difficulties [18]. Our goal was to investigate whether the discrepancy between non-verbal intelligence (RPM) and accuracy at reading emotional expressions in the eyes (RMET) would predict the magnitude of self-perceived social–emotional difficulties measured with the EQ in ASD and in controls.

## Participants and methods

### Sample description

All procedures were in accordance with the Declaration of Helsinki and were approved by the University Hospital ethics committee. Written informed consent was obtained from all adult participants and from all parents of participating adolescents. All adolescents also gave their oral assent. The total sample size was 107. Among those, 33 (3 females) were clinically diagnosed with ASD using DSM IV-TR criteria in combination with either the Autism Diagnostic Observation Schedule (ADOS [21]) and the Autism Diagnostic Interview-Revised (ADI-R [23] 1994), or the Diagnostic Interview for Social and Communication Disorder (DISCO [19]). None of the 74 controls (CON, 13 females) had a history of psychiatric/neurological disorders. See Table 1.

**Table 1:** Descriptive statistics of all the variables measured

Variable	ASD (*n* = 33,3F)	Controls (*n* = 74,13F)	Group difference
Age ± SD (range)	22.3 ± 7.91 (14.1–41.7)	22.9 ± 6.77 (14.1–41.9)	*p* = 0.703, *d* = − 0.083
RPM score ± SD (range)	10.2 ± 1.81 (5–12)	10.7 ± 1.30 (7–12)	*p* = 0.123, *d* = − 0.348
RMET score ± SD (range)	21.9 ± 4.87 (11–31)	24.9 ± 2.93 (18–31)	***p* = 0.002**, ***d* = **− **0.753**
EQ ± SD (range)	24.5 ± 8.35 (9–41)	38.9 ± 9.60 (23–67)	***p* < 0.001**, ***d* = **− **1.596**
Discrepancy score ± SD (range)	0.285 ± 1.60 (− 3.48 to 2.87)	− 0.127 ± 1.05 (− 3.02 to 2.25)	*p* = 0.183, *d* = 0.304

Significant differences are in bold

*RPM* Raven’s progressive matrices,* RMET* reading the mind in the eyes test, *EQ* empathy quotient, *d* Cohen’s effect size

### Statistical analyses

Differences between groups were performed using Welch’s *t* tests to account for the difference in sample size and in differential variance between the two groups. To compute the discrepancy between RPM and RMET scores, data were first z-transformed, and for each participant we computed the value of the discrepancy score [(z-RPM)-(z-RMET)]. A bivariate linear regression was conducted to examine how well the discrepancy could predict EQ for each group, separately.

## Results

Group differences are presented in Table 1. ASD and control participants did not differ in age or in IQ as assessed by the RPM. However, as expected, ASD had significantly lower scores on the RMET (*p* = 0.002, *d* = − 0.833) and EQ (*p* < 0.001, *d* = − 1.555). Groups did not differ in the discrepancy score computed as explained above (*p* = 0.183, *d* = 0.304).

### Linear regressions

#### Main analysis: discrepancy score and EQ (fig. 1)

For the ASD group, the correlation between discrepancy score and EQ was statistically significant, *r*(31) = 0.664, *p* < 0.001, with a large effect size [5]. The regression equation for predicting EQ was Y = 25.5–3.46*X. The *r*^2^ for this equation was 0.441; that is, 44.1% of the variance in EQ was predictable from the discrepancy score.

For the control group, the correlation between discrepancy score and EQ was not statistically significant, *r*(72) = 0.076, *p* = 0.522. The regression equation for predicting EQ was Y = 38.97 + 0.69*X. The *r*^2^ for this equation was 0.006; that is, only 0.6% of the variance in EQ was predictable from the discrepancy score.

Additional correlations were also examined.

**RPM and RMET**: The correlation between RPM and RMET score was not statistically significant for either group (ASD: *r*(31) = 0.159, *p* = 0.378; CON: *r*(72) = 0.170, *p* = 0.148).

**RMET and EQ:** The correlation between the RMET score and EQ was significant for the ASD group: *r*(31) = 0.379, *p* = 0.029. It was not significant for the control group: *r*(72) = 0.074, *p* = 0.528. The *r*^2^ for the regression equation was 0.144, meaning that 14.4% of the variance in EQ was explained by the RMET score in the ASD group, which is less than the 44.1% of the variance in EQ explained by the gap between the RPM and the RMET scores.

**RPM and EQ**: The correlation between RPM and EQ was also significant for the ASD group: *r*(31) = 0.484, *p* = 0.004, and it was not significant for the control group: *r*(72) = 0.156, *p* = 0.185. The *r*^2^ for the regression equation was 0.234, meaning that 23.4% of the variance in EQ was explained by RPM in the ASD group, which is less than the 44.1% of the variance in EQ explained by the gap between the RPM and the RMET scores.

**Fig. 1:**
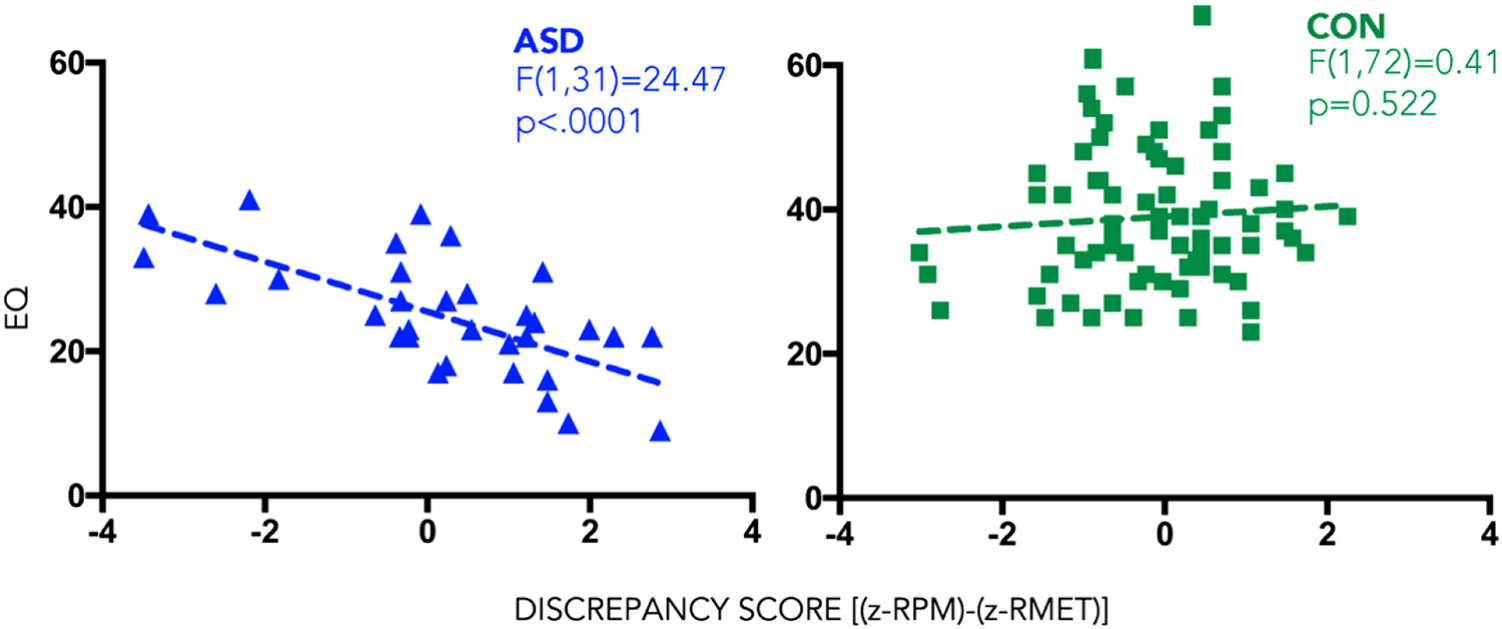
Linear regression between RPM-RMET discrepancy score and EQ in ASD (blue, left panel) and controls (green, right panel)

The gap score was, therefore, the best predictor of the variance in EQ (Fig. 1).

## Discussion

Causes of the poorer than expected outcome in a proportion of autistic individuals who do not have intellectual disability are still not well understood. Here, we show that contrary to what is observed in typical individuals, where the variability in socio-emotional processing cannot be explained by the difference between abstract reasoning and emotion understanding, autistic individuals report significantly more difficulties in cognitive empathy, emotional reactivity and social skills as the gap between these two constructs deepens. Our results suggests that it is specifically the magnitude of the gap between (high) levels of abstract reasoning skills and poor proficiency in reading emotions expressed by the eyes that predicts such self-perceived difficulties in emotional and social interactions among adults with autism. In terms of possible explanations, at an individual level, some autistic individuals find looking at the eyes of others aversive, as indicated by heightened amygdala activation during direct eye contact [12, 24, 30, 32], for review, see [29]. Because the eyes contain a lot of information regarding emotions and intentions, eye contact avoiding may result in reduced expertise, and in increased difficulties in social processing and emotional understanding.

## Limitations

We did not collect broader measures of adaptive behaviors such as the Vineland, QoL, or executive functions in this group of participants, and relied on self-reported questionnaire (EQ).

## Conclusion

The WHO defines QoL as “an individual’s perception of their position in life in the context of the culture and value systems in which they live and in relation to their goals, expectations, standards and concerns” [2]. A number of studies consistently report worse QoL in ASD_noID_ individuals (e.g., [7, 25]), but the underlying causes are not completely understood. We propose that one of the predictors of poorer than expected outcome in this population could be explained by the discrepancy between intact non-verbal intelligence and difficulties in socio-cognitive skills, that may be the result of eye avoidance used to self-regulate hyperarousal. There is a need for a more precise understanding of the relationship between issues related to social processing and/or to sensory processing and reduced QoL among autistic individuals, and how these may be experienced differently depending on cognitive capacities.

## Supplementary Information

Below is the link to the electronic supplementary material.

Supplementary file1 (XLSX 17 KB)
